# Na_7_Al_3_(As_2_O_7_)_4_

**DOI:** 10.1107/S1600536813020151

**Published:** 2013-07-31

**Authors:** Noura Fakhar Bourguiba, Ahmed Driss

**Affiliations:** a Laboratoire de Matériaux et Cristallochimie, Faculté des Sciences de Tunis, Université de Tunis El Manar, 2092 Manar II Tunis, Tunisia

## Abstract

The title compound, hepta­sodium trialuminium tetrakis(diarsenate), has been isolated as single crystals from a solid-state reaction. Its structure, which is isotypic with that of the Na_7_Fe_3_(*X*_2_O_7_)_4_ (*X* = As, P) family of compounds, consists of AlO_6_ octa­hedra sharing their vertices with As_2_O_7_ groups, forming a three-dimensional [Al_3_(As_2_O_7_)_4_]_∞_ framework incorporating channels occupied by the sodium ions. One of the aluminium ions lies on a crystallographic twofold axis. The sodium ions are situated over ten positions (one with site symmetry 2), all but one of which are partially occupied.

## Related literature

For isotopic compounds, see: Masquelier & d’Yvoire (1991 ▶); Masquelier *et al.* (1990 ▶, 1994 ▶); Quarez *et al.*(2009 ▶, 2010 ▶). For bond lengths and angles in related structures, see: Driss & Jouini (1994 ▶); Masquelier *et al.* (1995 ▶). For structural relationships, see: Lii *et al.* (1989 ▶); Hwu & Willis (1991 ▶); Boughzala *et al.* (1993) ▶; Boughzala & Jouini (1995 ▶); Lin & Lii (1996 ▶); Fukuoka *et al.* (2003 ▶); Ouerfelli *et al.* (2007 ▶). For bond-valence parameters, see: Brown & Altermatt (1985 ▶).

## Experimental

### 

#### Crystal data

Na_7_Al_3_(As_2_O_7_)_4_
*M* *_r_* = 1289.23
Monoclinic, 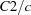

*a* = 9.800 (1) Å
*b* = 8.468 (1) Å
*c* = 28.637 (2) Å
β = 94.14 (1)°
*V* = 2370.3 (4) Å^3^
*Z* = 4
Mo *K*α radiation
μ = 11.50 mm^−1^
*T* = 298 K
0.25 × 0.20 × 0.10 mm

#### Data collection

Enraf–Nonius CAD-4 diffractometer
Absorption correction: ψ scan (North *et al.*, 1968 ▶) *T*_min_ = 0.759, *T*_max_ = 0.891
3958 measured reflections
2583 independent reflections
2150 reflections with *I* > 2σ(*I*)
*R*_int_ = 0.056
2 standard reflections every 120 min intensity decay: 1.1%

#### Refinement

*R*[*F* ^2^ > 2σ(*F* ^2^)] = 0.034
*wR*(*F* ^2^) = 0.085
*S* = 1.10
2583 reflections
221 parameters
Δρ_max_ = 1.27 e Å^−3^
Δρ_min_ = −0.98 e Å^−3^

### 

Data collection: *CAD-4 EXPRESS* (Duisenberg, 1992 ▶; Macíček & Yordanov, 1992 ▶); cell refinement: *CAD-4 EXPRESS*; data reduction: *XCAD4* (Harms & Wocadlo, 1995 ▶); program(s) used to solve structure: *SHELXS97* (Sheldrick, 2008 ▶); program(s) used to refine structure: *SHELXL97* (Sheldrick, 2008 ▶); molecular graphics: *DIAMOND* (Brandenburg, 1998 ▶); software used to prepare material for publication: *WinGX* (Farrugia, 2012 ▶).

## Supplementary Material

Crystal structure: contains datablock(s) I. DOI: 10.1107/S1600536813020151/hb7105sup1.cif

Structure factors: contains datablock(s) I. DOI: 10.1107/S1600536813020151/hb7105Isup2.hkl

Supplementary material file. DOI: 10.1107/S1600536813020151/hb7105Isup3.cml

Additional supplementary materials:  crystallographic information; 3D view; checkCIF report

## Figures and Tables

**Table 1:** Selected bond lengths (Å)

Al1—O12^i^	1.906 (4)
Al1—O12	1.906 (4)
Al1—O1^ii^	1.932 (4)
Al1—O1^iii^	1.932 (4)
Al1—O3	1.994 (4)
Al1—O3^i^	1.994 (4)
Al2—O14	1.920 (4)
Al2—O10^iv^	1.933 (4)
Al2—O7^iv^	1.942 (4)
Al2—O5	1.947 (4)
Al2—O9^v^	1.955 (4)
Al2—O6^ii^	2.000 (4)
As1—O2	1.651 (4)
As1—O1	1.676 (4)
As1—O3	1.689 (4)
As1—O4	1.776 (4)
As2—O13	1.638 (4)
As2—O14	1.675 (4)
As2—O12	1.686 (4)
As2—O4	1.769 (4)
As3—O7	1.668 (4)
As3—O5	1.671 (4)
As3—O6	1.677 (4)
As3—O11	1.736 (4)
As4—O8	1.646 (4)
As4—O10	1.676 (4)
As4—O9	1.679 (4)
As4—O11	1.760 (4)

Symmetry codes: (i) 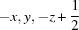; (ii) 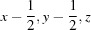; (iii) 
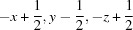
; (iv) 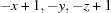; (v) 
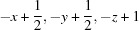
.

**Fig. 1.**
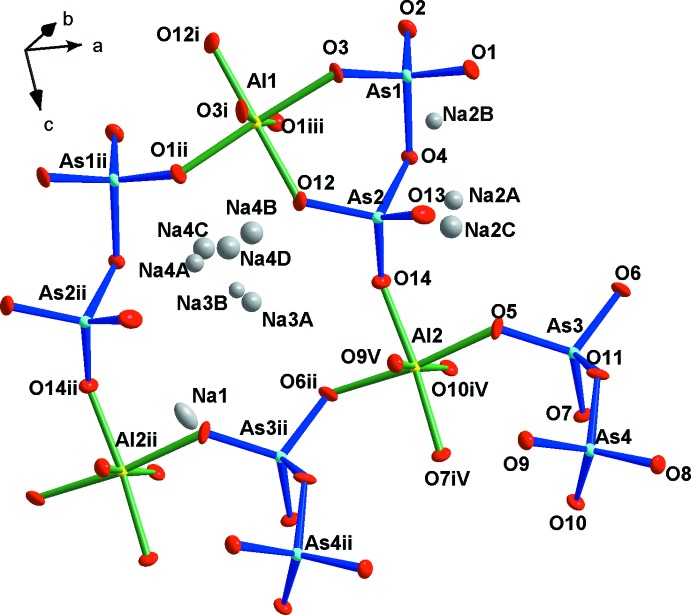
Part of Na7Al3 (As2O7) 4 structure. The ellipsoids were defined with 50% probability. [Symmetry code: (i): - x, y, - z + 0.5; (ii): x - 1/2, y - 1/2, z; (iii): - x + 1/2, y - 1/2, - z + 0.5; (iv): - x + 1, - y, - z + 1; (v): -x + 1/2, - y + 1/2, - z + 1].

**Fig. 2.**
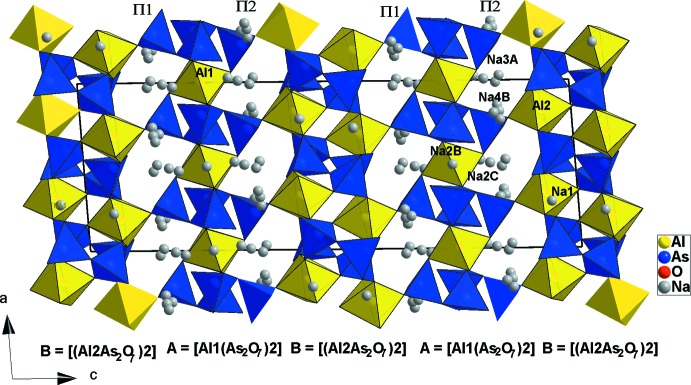
Projection of Na7Al3(As2O7)4 structure along [010]. The Na sites are numbered. The picture indicates the location of the A and B layers and the π planes at z≈ 0.15 and 0.35.

**Fig. 3.**
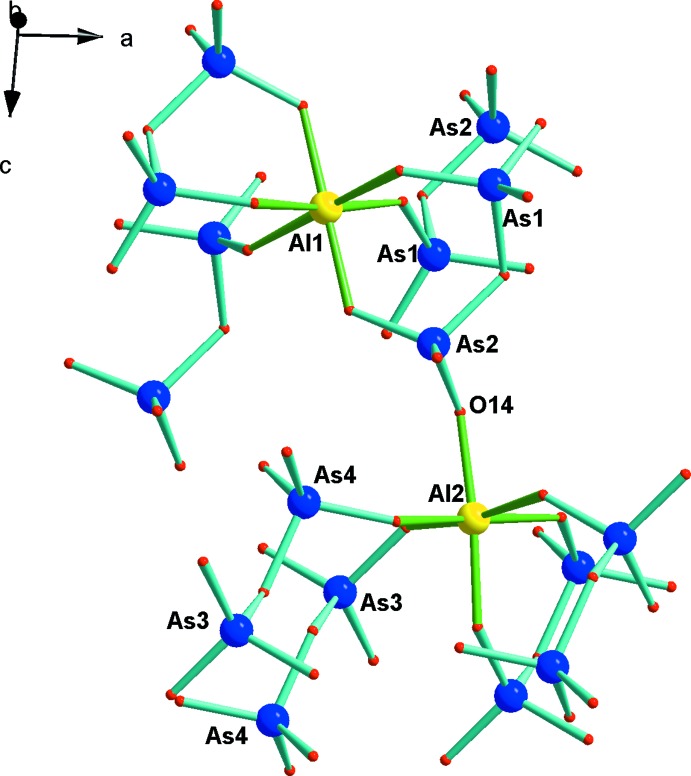
Arrangement of Al1O6 octahedra and As1As2O7 groups in the A-layer and arrangement of Al2O6 octahedra and As3As4O7 groups in the B-layer.

**Fig. 4.**
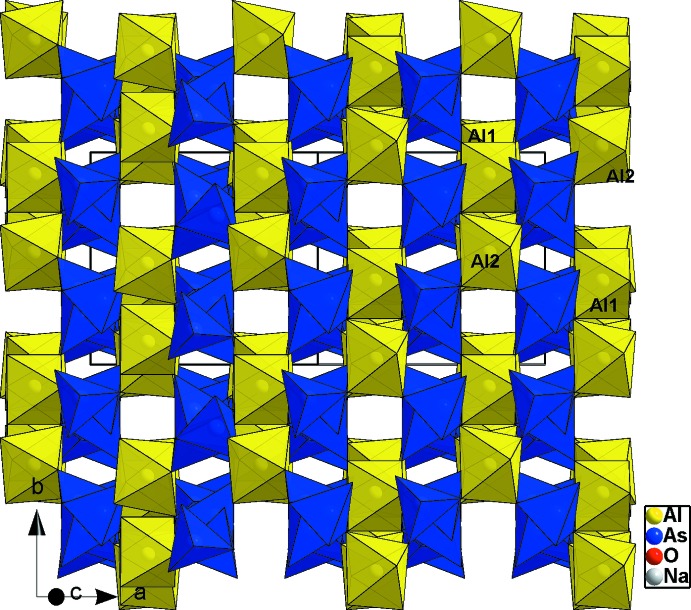
Alternate stacking of octahedral AlO6 layers and As2O7 groups layers parallel to (-101).

## supplementary crystallographic information

### Comment

A new family of compounds Na_7_*M*_3_(*X*_2_O_7_)_4_ (*M* = Fe, Al, Cr, Ga; *X* = P, As) studied by Masquelier *et al.* (1994) present interesting ion conductivity properties. In this family line, a structural study was carried out only for Na_7_Fe_3_(As_2_O_7_)_4_ and Na_7_Fe_3_(P_2_O_7_)_4_ compounds by Masquelier *et al.*(1990, 1991). In addition, another structural study was conducted to isotypes compounds Na_5_Ag_2_Fe_3_(As_2_O_7_)_4_ and Na_2_Ag_5_Fe_3_(P_2_O_7_)_4_ by Quarez *et al.* (2009). Also, electrical measurements were also made by Quarez *et al.* (2009) for Ag_7_Fe_3_(*X*_2_O_7_)_4_ (*X* = As, P) compounds. However, as for the Na_7_Al_3_(As_2_O_7_)_4_ compound no structural analysis has been reported so far.

The structure of compound Na_7_Al_3_(As_2_O_7_)_4_ shows a three-dimensional anionic framework [Al_3_(As_2_O_7_) _4_]_∞_ composed of octahedron AlO_6_ sharing vertices with As_2_O_7_ groups. This framework defines the interstitial spaces in which the Na ^+^ ions are located in a partially disordered distribution to ensure the electrical neutrality. The single unit [Al_3_(As_2_O_7_)_4_]^7-^ consists of an octahedron Al1O_6_ and two octahedra Al2O_6_ related to As_2_O_7_ group by sharing vertices (Fig. 1). The two aluminium atoms occupy two crystallographically independent sites: the aluminium Al1 is in special position 4e of a symmetry equal to 2; whereas the Al2 is in general position of 8f. In this unit, the connection between the octahedra Al1O_6_ and Al2O_6_ is provided by the group As1As2O_7_ through oxygen atom O14. In this structure, the units [Al_3_(As_2_O_7_)_4_]^7-^ are inter-related thanks to composite bridges of types Al—O—As and As—O—Al respectively. These bridges are located between the octahedra AlO_6_ and As_2_O_7_ diarsenates groups in order to handle two types of parallel layers to the (a, b) plan. According to (010) (Fig. 2), a projection of the structure shows that the anionic framework consists of a succession of layers as follow: A: [Al1(As2As3O_7_)_2_]_∞_ and B: [(Al2As3As4O_7_)_2_]_∞_ perpendicular to c alternately. In the layers A, the only components are Octahedra Al1O_6_, whereas other aluminium octahedra Al2O_6_ shape layers B.

On the one hand, in the layer A, each octahedron Al1O_6_ is connected to two groups As1As2O_7_ by sharing both a vertex and two other groups As1As2O_7_ such as the Al1O_6_ octahedron shares two of its vertices with the same group As_2_O_7_. By rotation around the fold axis, these groups are equivalent (Fig. 3). This mode of connection is found in phosphates: ARu_2_(P_2_O_7_)_2_ (A = Li, Na, Ag) (Fukuoka *et al.*, 2003.), NaMo_2_(P_2_O_7_)_2_ (Hwu *et al.*, 1991), and SrV_2_(P_2_O_7_)_2_ (Lii *et al.*, 1989).

On the other hand, in the layer B, each octahedron Al2O_6_ is connected to four As_2_O_7_ groups through the formation of composite bridges Al—O—As and the division of two vertices with a fifth group As_2_O_7_ (Fig. 3). This layer is formed by AlAs_2_O_11_ units related through the formation of Al—O—As composed bridges. This type of M—O—As bond between MAs_2_O_11_ units (*M* = Al, Ga, Fe) is situated in the following isostructural compounds: RbAlAs_2_O_7_ (Boughzala *et al.*, 1993), KAlAs_2_O_7_ (Boughzala *et al.*, 1995), KGaAs_2_O_7_ (Lin *et al.*, 1996) and TlFe_0.22_Al_0.78_As_2_O_7_ (Ouerfelli *et al.*, 2007).

The O14 oxygen atom guarantees the bond between the layers A and B through the bridges Al2—O14—As2.

The anionic framework [Al_3_(As_2_O_7_)_4_]_∞_ can be also described as alternate stacking of octahedral AlO_6_ layers and As_2_O_7_ groups layers parallel to (101) (Fig. 4).

In this structure, the sodium ions are distributed through ten crystallographic sites in the interstices of the anionic network. The first type of the site corresponding to 8f position is totally occupied by the Na1 cation which is localized in the layer B. As for the nine other sites, they are partially occupied. And these can be arranged into groups of three sets: (Na2A—Na2B—Na2C), (Na3A—Na3B) and (Na4A—Na4B—Na4C—Na4D). Different sites of the same group are too close to be occupied simultaneously. As a result, the total number of Na^+^ ions for each group is less than or equal to one. These sodium ions are predominantly located in the layer A and they are distributed in the vicinity of two plans parallel to (001) respectively to dimensions *z*≈ 0.15 and *z*≈ 0.35. Also, the Na2B site, located on the binary axis, occupies an intermediate position (*z* = 1/4). Examination of geometric factors in the structure shows that they are in accordance with those found in the literature (Driss *et al.*, 1994; Masquelier *et al.*, 1995.). In addition, the use of the BVS method for the calculation of different valences of bonds, using the empirical formula of Brown (Brown & Altermatt, 1985), does satisfy the expected values of ion charge: Al1 (2.73), Al2 (2.69), As1 (4.90), As2 (4.98), As3 (5.01), As4 (5.00), Na1 (1.16), Na2A (0.87), Na2B (1.13), Na2C (0.79), Na3A (1.00), Na3B (1.03), Na4A (0.97), Na4B (0.95), Na4C (1.01), Na4D (0, 93). In order to use these structural data and to connect them to the physicochemical properties, especially ion-conducting electrical measurements through a complex impedance bridge are in progress.

### Experimental

The crystals related to the Na_7_Al_3_ (As_2_O_7_)_4_ phase were obtained from reactants: NaHCO_3_ (Prolabo, 27778), Al_2_O_3_ (Riedel-de Haen, 167305) NH_4_H_2_AsO_4_ (prepared in the laboratory, JCPDS-775), taken in molar proportions: Na: A l: As = 10: 1: 9. Finely ground, the mixture was put in a porcelain crucible, placed in an oven and preheated in air at 673 K for 24 h to remove volatiles. After that, the mixture is raised to a synthesis temperature close to the melting 968 K through stages of 100 degrees followed by grinding. The mixture is then left at this temperature for a week to promote germination and growth of crystals. The final residue undergoes a first slow cooling (5 ° / half day, at 800 K) and a second fast (50 ° / h) to room temperature. Transparent crystals of prismatic form, clear outline and sufficient size for measurements of intensities, have been separated from the stream by successive hot water washes.

### Refinement

The collection was carried out in the monoclinic system of C2 / c space group. During the final refinement and for electrical neutrality reasons, the occupancy rate of Na^+^ cations were conducted using the SUMP condition authorized by the *SHELXL* program. The refinement of all variable parameters leads to well defined ellipsoids. The maximum and minimum densities of electrons remaining in the Fourier-difference are respectively situated at 0.86 Å from the As4 site and at 1.35 Å from the NA3B site.

### Crystal data

**Table:**

Na_7_Al_3_(As_2_O_7_)_4_	*F*(000) = 2416
*M**_r_* = 1289.23	*D*_x_ = 3.613 Mg m^−^^3^
Monoclinic, *C*2/*c*	Mo *K*α radiation, λ = 0.71073 Å
Hall symbol: -C 2yc	Cell parameters from 25 reflections
*a* = 9.800 (1) Å	θ = 10–15°
*b* = 8.468 (1) Å	µ = 11.50 mm^−^^1^
*c* = 28.637 (2) Å	*T* = 298 K
β = 94.14 (1)°	Parallelipiped, colorless
*V* = 2370.3 (4) Å^3^	0.25 × 0.20 × 0.1 mm
*Z* = 4	

### Data collection

**Table:**

Enraf–Nonius CAD-4 diffractometer	2150 reflections with *I* > 2σ(*I*)
Radiation source: fine-focus sealed tube	*R*_int_ = 0.056
Graphite monochromator	θ_max_ = 27.0°, θ_min_ = 2.9°
ω/2θ scans	*h* = −12→3
Absorption correction: ψ scan (North *et al.*, 1968)	*k* = −1→10
*T*_min_ = 0.759, *T*_max_ = 0.891	*l* = −36→36
3958 measured reflections	2 standard reflections every 120 min
2583 independent reflections	intensity decay: 1.1%

### Refinement

**Table:**

Refinement on *F*^2^	Primary atom site location: structure-invariant direct methods
Least-squares matrix: full	Secondary atom site location: difference Fourier map
*R*[*F*^2^ > 2σ(*F*^2^)] = 0.034	*w* = 1/[σ^2^(*F*_o_^2^) + (0.0314*P*)^2^ + 24.9095*P*] where *P* = (*F*_o_^2^ + 2*F*_c_^2^)/3
*wR*(*F*^2^) = 0.085	(Δ/σ)_max_ = 0.001
*S* = 1.10	Δρ_max_ = 1.27 e Å^−^^3^
2583 reflections	Δρ_min_ = −0.98 e Å^−^^3^
221 parameters	Extinction correction: *SHELXL97* (Sheldrick, 2008), Fc^*^=kFc[1+0.001xFc^2^λ^3^/sin(2θ)]^-1/4^
0 restraints	Extinction coefficient: 0.00057 (6)

### Special details

**Table:**

Geometry. All e.s.d.'s (except the e.s.d. in the dihedral angle between two l.s. planes) are estimated using the full covariance matrix. The cell e.s.d.'s are taken into account individually in the estimation of e.s.d.'s in distances, angles and torsion angles; correlations between e.s.d.'s in cell parameters are only used when they are defined by crystal symmetry. An approximate (isotropic) treatment of cell e.s.d.'s is used for estimating e.s.d.'s involving l.s. planes.
Refinement. Refinement of *F*^2^ against ALL reflections. The weighted *R*-factor *wR* and goodness of fit *S* are based on *F*^2^, conventional *R*-factors *R* are based on *F*, with *F* set to zero for negative *F*^2^. The threshold expression of *F*^2^ > σ(*F*^2^) is used only for calculating *R*-factors(gt) *etc*. and is not relevant to the choice of reflections for refinement. *R*-factors based on *F*^2^ are statistically about twice as large as those based on *F*, and *R*- factors based on ALL data will be even larger.

### Fractional atomic coordinates and isotropic or equivalent isotropic displacement parameters (Å^2^)

**Table:**

	*x*	*y*	*z*	*U*_iso_*/*U*_eq_	Occ. (<1)
Al1	0.0000	0.1057 (3)	0.2500	0.0082 (4)	
Al2	0.31736 (15)	−0.02622 (18)	0.43636 (5)	0.0069 (3)	
As1	0.30056 (5)	0.28137 (6)	0.246216 (18)	0.00898 (15)	
As2	0.21753 (5)	0.15080 (6)	0.338657 (18)	0.00927 (15)	
As3	0.58680 (5)	0.20071 (6)	0.464306 (18)	0.00733 (14)	
As4	0.48861 (5)	0.34009 (6)	0.557696 (18)	0.00774 (14)	
O1	0.3645 (4)	0.4537 (5)	0.26651 (14)	0.0149 (8)	
O2	0.3734 (4)	0.2071 (5)	0.20087 (13)	0.0149 (8)	
O3	0.1281 (4)	0.2823 (5)	0.23865 (14)	0.0148 (8)	
O4	0.3327 (4)	0.1491 (4)	0.29386 (13)	0.0119 (8)	
O5	0.4404 (4)	0.1537 (5)	0.43395 (15)	0.0170 (9)	
O6	0.6943 (4)	0.2857 (5)	0.42888 (14)	0.0145 (8)	
O7	0.6569 (4)	0.0529 (4)	0.49622 (13)	0.0120 (8)	
O8	0.5686 (4)	0.4760 (5)	0.59037 (13)	0.0148 (8)	
O9	0.3192 (4)	0.3690 (5)	0.54893 (14)	0.0142 (8)	
O10	0.5262 (4)	0.1554 (4)	0.57538 (14)	0.0133 (8)	
O11	0.5459 (4)	0.3542 (4)	0.50101 (13)	0.0139 (8)	
O12	0.0599 (4)	0.1063 (5)	0.31482 (14)	0.0162 (8)	
O13	0.2283 (5)	0.3289 (5)	0.36098 (15)	0.0210 (9)	
O14	0.2752 (4)	−0.0052 (5)	0.37014 (13)	0.0162 (8)	
Na1	−0.2221 (3)	−0.1225 (3)	0.44633 (9)	0.0249 (6)	
Na2A	0.5268 (10)	−0.0466 (12)	0.3075 (4)	0.017 (2)*	0.24
Na2B	0.5000	0.0010 (13)	0.2500	0.013 (2)*	0.28
Na2C	0.5094 (8)	−0.0556 (10)	0.3269 (3)	0.0236 (18)*	0.32
Na3A	0.0283 (5)	−0.1551 (6)	0.35860 (17)	0.0186 (11)*	0.58
Na3B	−0.0112 (8)	−0.1372 (9)	0.3504 (3)	0.0118 (16)*	0.33
Na4A	−0.233 (2)	0.100 (3)	0.3543 (7)	0.016 (4)*	0.13
Na4B	−0.1483 (11)	0.2292 (14)	0.3505 (3)	0.024 (2)*	0.27
Na4C	−0.1939 (9)	0.0862 (10)	0.3420 (3)	0.0220 (17)*	0.35
Na4D	−0.184 (2)	0.175 (3)	0.3538 (7)	0.025 (4)*	0.14

### Atomic displacement parameters (Å^2^)

**Table:**

	*U*^11^	*U*^22^	*U*^33^	*U*^12^	*U*^13^	*U*^23^
Al1	0.0067 (9)	0.0083 (10)	0.0094 (10)	0.000	0.0000 (8)	0.000
Al2	0.0071 (7)	0.0064 (7)	0.0072 (7)	0.0000 (6)	−0.0004 (5)	0.0007 (6)
As1	0.0065 (2)	0.0108 (3)	0.0096 (3)	−0.00147 (19)	0.00072 (18)	0.00172 (19)
As2	0.0100 (3)	0.0103 (3)	0.0074 (3)	−0.0014 (2)	0.00003 (18)	0.0014 (2)
As3	0.0066 (2)	0.0081 (3)	0.0074 (3)	0.00032 (19)	0.00102 (18)	0.00067 (19)
As4	0.0076 (2)	0.0075 (3)	0.0083 (3)	−0.00002 (19)	0.00122 (18)	−0.00046 (18)
O1	0.0154 (19)	0.0102 (19)	0.019 (2)	−0.0034 (16)	0.0032 (15)	−0.0008 (16)
O2	0.0134 (18)	0.017 (2)	0.0151 (19)	−0.0001 (16)	0.0077 (14)	−0.0027 (16)
O3	0.0036 (16)	0.016 (2)	0.025 (2)	−0.0003 (15)	0.0023 (14)	0.0049 (17)
O4	0.0077 (16)	0.0145 (19)	0.0136 (18)	0.0026 (15)	0.0024 (14)	0.0037 (15)
O5	0.0125 (18)	0.0120 (19)	0.025 (2)	−0.0018 (15)	−0.0103 (16)	−0.0004 (16)
O6	0.0127 (18)	0.017 (2)	0.0149 (19)	0.0003 (16)	0.0066 (14)	0.0055 (16)
O7	0.0139 (18)	0.0114 (19)	0.0103 (18)	0.0063 (15)	−0.0011 (14)	0.0018 (15)
O8	0.0169 (19)	0.0147 (19)	0.013 (2)	−0.0044 (16)	0.0005 (15)	−0.0055 (15)
O9	0.0089 (17)	0.0145 (19)	0.019 (2)	0.0014 (15)	0.0009 (15)	0.0017 (16)
O10	0.0145 (18)	0.0076 (18)	0.018 (2)	0.0010 (15)	0.0025 (15)	0.0047 (15)
O11	0.025 (2)	0.0084 (18)	0.0088 (18)	0.0017 (16)	0.0077 (15)	0.0018 (15)
O12	0.0063 (16)	0.024 (2)	0.018 (2)	−0.0019 (16)	−0.0012 (14)	0.0011 (17)
O13	0.035 (2)	0.0112 (19)	0.017 (2)	−0.0013 (18)	0.0032 (17)	−0.0012 (17)
O14	0.022 (2)	0.0135 (19)	0.013 (2)	−0.0012 (16)	−0.0021 (15)	0.0030 (16)
Na1	0.0255 (13)	0.0141 (12)	0.0370 (15)	−0.0030 (10)	0.0148 (11)	−0.0035 (11)

### Geometric parameters (Å, º)

**Table:**

Al1—O12^i^	1.906 (4)	Na2B—O3^ix^	2.274 (10)
Al1—O12	1.906 (4)	Na2B—O3^iii^	2.274 (10)
Al1—O1^ii^	1.932 (4)	Na2B—O4^viii^	2.477 (7)
Al1—O1^iii^	1.932 (4)	Na2B—O2^viii^	2.512 (9)
Al1—O3	1.994 (4)	Na2C—O13^ix^	2.492 (9)
Al1—O3^i^	1.994 (4)	Na2C—O3^iii^	2.619 (9)
Al2—O14	1.920 (4)	Na2C—O2^viii^	2.652 (10)
Al2—O10^iv^	1.933 (4)	Na2C—O12^ix^	2.931 (9)
Al2—O7^iv^	1.942 (4)	Na2C—O10^iv^	2.968 (11)
Al2—O5	1.947 (4)	Na3A—O2^iii^	2.330 (6)
Al2—O9^v^	1.955 (4)	Na3A—O8^v^	2.353 (6)
Al2—O6^ii^	2.000 (4)	Na3A—O6^ii^	2.545 (7)
As1—O2	1.651 (4)	Na3A—O5^ii^	2.879 (6)
As1—O1	1.676 (4)	Na3A—O13^ii^	2.949 (7)
As1—O3	1.689 (4)	Na3B—O8^v^	2.279 (8)
As1—O4	1.776 (4)	Na3B—O2^iii^	2.451 (8)
As2—O13	1.638 (4)	Na3B—O13^ii^	2.608 (9)
As2—O14	1.675 (4)	Na3B—O1^ii^	2.725 (9)
As2—O12	1.686 (4)	Na3B—O4^ii^	2.807 (8)
As2—O4	1.769 (4)	Na3B—O6^ii^	2.980 (9)
As3—O7	1.668 (4)	Na4A—O2^i^	2.213 (19)
As3—O5	1.671 (4)	Na4A—O8^v^	2.271 (19)
As3—O6	1.677 (4)	Na4A—O13^ii^	2.33 (2)
As3—O11	1.736 (4)	Na4A—O6^vii^	2.79 (2)
As4—O8	1.646 (4)	Na4B—O14^x^	2.449 (11)
As4—O10	1.676 (4)	Na4B—O8^v^	2.512 (11)
As4—O9	1.679 (4)	Na4B—O10^v^	2.555 (11)
As4—O11	1.760 (4)	Na4B—O2^i^	2.568 (11)
Na1—O8^v^	2.265 (5)	Na4B—O3^i^	2.613 (11)
Na1—O9^vi^	2.302 (5)	Na4B—O6^vii^	2.854 (11)
Na1—O7^vii^	2.429 (4)	Na4C—O8^v^	2.278 (8)
Na1—O13^ii^	2.492 (5)	Na4C—O2^i^	2.310 (9)
Na1—O5^ii^	2.517 (5)	Na4C—O13^ii^	2.384 (9)
Na1—O9^ii^	2.938 (5)	Na4C—O1^ii^	2.538 (10)
Na1—O11^ii^	2.966 (5)	Na4C—O3^i^	2.954 (9)
Na1—O10^vi^	3.013 (5)	Na4D—O8^v^	2.28 (2)
Na2A—O2^viii^	2.380 (11)	Na4D—O2^i^	2.36 (2)
Na2A—O3^iii^	2.420 (10)	Na4D—O6^vii^	2.70 (2)
Na2A—O13^ix^	2.631 (11)	Na4D—O14^x^	2.78 (3)
Na2A—O3^ix^	2.692 (12)	Na4D—O10^v^	2.85 (2)
Na2A—O12^ix^	2.962 (11)	Na4D—O3^i^	2.89 (2)
			
O12^i^—Al1—O12	179.7 (3)	O2—As1—O4	107.54 (19)
O12^i^—Al1—O1^ii^	94.10 (18)	O1—As1—O4	103.99 (19)
O12—Al1—O1^ii^	86.13 (17)	O3—As1—O4	102.82 (18)
O12^i^—Al1—O1^iii^	86.13 (17)	O13—As2—O14	120.4 (2)
O12—Al1—O1^iii^	94.10 (18)	O13—As2—O12	113.3 (2)
O1^ii^—Al1—O1^iii^	96.5 (3)	O14—As2—O12	107.8 (2)
O12^i^—Al1—O3	89.56 (18)	O13—As2—O4	105.3 (2)
O12—Al1—O3	90.19 (17)	O14—As2—O4	100.13 (19)
O1^ii^—Al1—O3	172.35 (19)	O12—As2—O4	108.58 (18)
O1^iii^—Al1—O3	90.44 (16)	O7—As3—O5	114.24 (19)
O12^i^—Al1—O3^i^	90.19 (17)	O7—As3—O6	113.67 (19)
O12—Al1—O3^i^	89.56 (18)	O5—As3—O6	109.9 (2)
O1^ii^—Al1—O3^i^	90.44 (16)	O7—As3—O11	109.49 (18)
O1^iii^—Al1—O3^i^	172.35 (19)	O5—As3—O11	105.4 (2)
O3—Al1—O3^i^	82.8 (2)	O6—As3—O11	103.15 (19)
O14—Al2—O10^iv^	89.86 (18)	O8—As4—O10	113.3 (2)
O14—Al2—O7^iv^	174.86 (18)	O8—As4—O9	114.1 (2)
O10^iv^—Al2—O7^iv^	93.40 (17)	O10—As4—O9	111.99 (19)
O14—Al2—O5	88.99 (18)	O8—As4—O11	107.83 (19)
O10^iv^—Al2—O5	86.32 (18)	O10—As4—O11	105.32 (18)
O7^iv^—Al2—O5	95.18 (18)	O9—As4—O11	103.31 (19)
O14—Al2—O9^v^	92.74 (18)	As1—O1—Al1^xi^	137.9 (2)
O10^iv^—Al2—O9^v^	170.74 (18)	As1—O3—Al1	127.7 (2)
O7^iv^—Al2—O9^v^	84.66 (17)	As2—O4—As1	117.7 (2)
O5—Al2—O9^v^	84.85 (18)	As3—O5—Al2	132.7 (2)
O14—Al2—O6^ii^	83.26 (17)	As3—O6—Al2^xi^	132.6 (2)
O10^iv^—Al2—O6^ii^	90.59 (17)	As3—O7—Al2^iv^	130.6 (2)
O7^iv^—Al2—O6^ii^	92.73 (17)	As4—O9—Al2^v^	138.8 (2)
O5—Al2—O6^ii^	171.67 (19)	As4—O10—Al2^iv^	129.4 (2)
O9^v^—Al2—O6^ii^	98.54 (17)	As3—O11—As4	127.6 (2)
O2—As1—O1	115.63 (19)	As2—O12—Al1	127.1 (2)
O2—As1—O3	112.7 (2)	As2—O14—Al2	130.2 (2)
O1—As1—O3	112.73 (19)		

Symmetry codes: (i) −*x*, *y*, −*z*+1/2; (ii) *x*−1/2, *y*−1/2, *z*; (iii) −*x*+1/2, *y*−1/2, −*z*+1/2; (iv) −*x*+1, −*y*, −*z*+1; (v) −*x*+1/2, −*y*+1/2, −*z*+1; (vi) −*x*, −*y*, −*z*+1; (vii) *x*−1, *y*, *z*; (viii) −*x*+1, *y*, −*z*+1/2; (ix) *x*+1/2, *y*−1/2, *z*; (x) *x*−1/2, *y*+1/2, *z*; (xi) *x*+1/2, *y*+1/2, *z*.

## References

[bb1] Boughzala, H., Driss, A. & Jouini, T. (1993). *Acta Cryst.* C**49**, 425–427.

[bb2] Boughzala, H. & Jouini, T. (1995). *Acta Cryst.* C**51**, 179–181.

[bb3] Brandenburg, K. (1998). *DIAMOND* University of Bonn, Germany.

[bb4] Brown, I. D. & Altermatt, D. (1985). *Acta Cryst.* B**41**, 244–247.

[bb5] Driss, A. & Jouini, T. (1994). *J. Solid State Chem.* **112**, 277–280.

[bb6] Duisenberg, A. J. M. (1992). *J. Appl. Cryst.* **25**, 92–96.

[bb7] Farrugia, L. J. (2012). *J. Appl. Cryst.* **45**, 849–854.

[bb8] Fukuoka, H., Matsunaga, H. & Yamanaka, S. (2003). *Mater. Res. Bull.* **38**, 991–1001.

[bb9] Harms, K. & Wocadlo, S. (1995). *XCAD4* University of Marburg, Germany.

[bb10] Hwu, S.-J. & Willis, E. D. (1991). *J. Solid State Chem.* **93**, 69–76.

[bb11] Lii, K. H., Chen, J. J. & Wang, S. L. (1989). *J. Solid State Chem.* **78**, 178–183.

[bb12] Lin, K.-J. & Lii, K.-H. (1996). *Acta Cryst.* C**52**, 2387–2389.

[bb13] Macíček, J. & Yordanov, A. (1992). *J. Appl. Cryst.* **25**, 73–80.

[bb14] Masquelier, C. & d’Yvoire, F. (1991). *J. Solid State Chem.* **95**, 156–167.

[bb15] Masquelier, C., d’Yvoire, F., Bretey, E., Berthet, P. & Peytour-Chansac, C. (1994). *Solid State Ionics*, **67**, 183–189.

[bb16] Masquelier, C., d’Yvoire, F. & Collin, G. (1995). *J. Solid State Chem.* **118**, 33–44.

[bb17] Masquelier, C., d’Yvoire, F. & Rodier, N. (1990). *Acta Cryst.* C**46**, 1584–1587.

[bb18] North, A. C. T., Phillips, D. C. & Mathews, F. S. (1968). *Acta Cryst.* A**24**, 351–359.

[bb19] Ouerfelli, N., Guesmi, A., Mazza, D., Madani, A., Zid, M. F. & Driss, A. (2007). *J. Solid State Chem.* **180**, 1224–1229.

[bb20] Quarez, E., Mentré, O., Oumellala, Y. & Masquelier, C. (2009). *New J. Chem.* **33**, 998–1005.

[bb21] Quarez, E., Mentré, O., Oumellala, Y. & Masquelier, C. (2010). *New J. Chem.* **34**, 287–293.

[bb22] Sheldrick, G. M. (2008). *Acta Cryst.* A**64**, 112–122. 10.1107/S0108767307043930 18156677

